# Comparing a 7-day diary vs. 24 h-recall for estimating fluid consumption in overweight and obese Mexican women

**DOI:** 10.1186/s12889-015-2367-0

**Published:** 2015-10-07

**Authors:** Sonia Hernández-Cordero, Nancy López-Olmedo, Sonia Rodríguez-Ramírez, Simón Barquera-Cervera, Juan Rivera-Dommarco, Barry Popkin

**Affiliations:** Center of Research in Nutrition and Health, National Institute of Public Health, Av. Universidad 655 Col. Sta. Maria Ahuacatitlán, Cuernavaca, Morelos CP 62100 Mexico; Carolina Population Center, University of North Carolina at Chapel Hill, 123 W. Franklin St., Chapel Hill, NC 27516 USA

**Keywords:** Fluid intake measurement, Validation, Sugar-sweetened beverages, Dietary intake

## Abstract

**Background:**

High intake of sugar-sweetened beverages (SSB) is linked to increased weight, energy intake, and diabetes. Even though the increasing interest on beverages and water intake, there are few dietary tools carefully validated. The purpose of this paper is to compare a fluid intake 7-day diary against a 24-h recall questionnaire to estimate the fluid consumption in overweight and obese women participating in a randomized controlled trial in Mexico.

**Methods:**

This cross-sectional study explored the correlation of reported fluid consumption between two methods: 3-day 24-hr recalls and 7-day diary beverage registry in overweight and obese Mexican women aged 18–45 y (*n* = 190).

**Results:**

There was no difference on median estimated volume (mL/d), nor the median estimated energy (kcal/d) from total beverage consumption registered by the two dietary tools. The crude and rank correlation among the two dietary instruments was high for total fluid consumption in mL/d *r* = 0.7, *p* < 0.001 (crude and rank correlation) and for fluid consumption measured as energy intake: *r* = 0.7; *p* < 0.001 crude, and *r* = 0.5; *p* < 0.001 rank correlation. By type of beverage, the more meaningful rank correlations were for fluid intake in: mL/d, water, alcohol beverages, and SSB; and in kcal/d, alcohol beverages and SSBs (rank correlation ≥ 0.6).

**Conclusions:**

Overall, the 7-day diary showed high and strong rank correlations with that reported in the 24-h recall, suggesting that the diary method is a valid dietary tool to evaluate total fluid, water and SSB intake in this population.

**Electronic supplementary material:**

The online version of this article (doi:10.1186/s12889-015-2367-0) contains supplementary material, which is available to authorized users.

## Background

Caloric beverages, particularly sugar-sweetened beverages (SSB), represent an increasing component of our diet. Mattes and many others have shown that there are differential effects of calories consumed as a liquid beverage and a food [1–7]. In Mexico, the United States, the United Kingdom, Europe, and elsewhere increasing concern has focused on several key components of caloric beverages, those high in refined sugars, high-fructose corn syrup, or fruit juice concentrates [8–14]. Meta-analyses have provided evidence that high intake of SSB (e.g., soft drinks, fruit drinks) are linked to increased weight, energy intake, and diabetes [15–17]. Additional concern is raised about consumption of other beverages such as fruit juices, alcohol, and high-fat milk due to their contribution to energy intake and its association with obesity and chronic diseases such as type 2 diabetes and dyslipidemia, among others [18–26]. Some countries have developed programs and policies to promote water intake and limit access to SSB in schools and feeding programs, mainly through banning SSB within schools, and to restrict media related to SSB through regulation of the marketing of foods (especially to children). [27–29].

Mexico has experienced rapid increases in SSB and caloric intake from beverages in the last 20 years [12–14]. Furthermore, the government created a beverage guidance panel [30] and recently moved to reduce caloric intake from beverages in all age groups by taxing SSB [31]. In the Mexican government recommendations and those discussed by many other countries, water intake has been viewed as an important, viable alternative to these caloric beverages; however, there is limited information on water consumption patterns in the context of a total beverage intake pattern [11, 32–34].

There are no entirely objective measures of an individual’s food or drink consumption except in the controlled conditions of a metabolic unit. Any self-reported measurement of intake will be biased in some way by the measurement process itself. There are two main approaches to individual assessment: prospective and retrospective, each with their advantages and disadvantages [35].

Even though the increasing interest on beverages and water intake, there are few dietary tools carefully validated for U.S. adolescents [36] and adults [37–40]. To date no specific dietary instrument for simple collection of the full array of fluid has been carefully validated for Mexicans. For epidemiological studies, retrospective methods are usually applied. A standard 24-h recall or food frequency questionnaire is the most used; both focus on obtaining data on foods and beverages containing calories and macro- and micro-nutrients. [41]. For intervention and epidemiological studies, the use of a standard 24-h recall is more common, and the 24-h urine collection could be used as a marker of fluid intake [42], but collecting 24-h urine is not practical and therefore not always used in large epidemiological studies. The purpose of this paper is to compare a prospective method: a fluid intake 7-day diary against a retrospective one: 24-h recall questionnaire for 3 days to estimate the fluid consumption in overweight and obese Mexican women participating in a clinical trial to reduce SSBs and increase water consumption.

## Methods

### Subjects

Women participating in a randomized clinical trial with the primary objective to investigate whether replacement of SSBs with water could reduce plasma triglyceride concentrations and other cardiometabolic factors over 9 months in overweight and obese young Mexican women. Women were overweight or obese (body mass index [BMI] range ≥25 kg/m^2^ and <39 kg/m^2^), aged 18–45 y, and otherwise healthy. Eligible participants reported consuming at least 250 kcal per day (kcal/d) of caloric beverages (e.g., includes soft drinks, juices-100 % juices and fruit drinks-sport drinks, sweetened tea or coffee, and alcoholic beverages) measured through a 24-h recall applied three randomly selected nonconsecutive days during one week (two weekdays and one weekend). As part of the original study, anthropometry, diet, physical activity, blood pressure, and fasting blood were collected at baseline and at 3, 6, and 9 months. In addition, women were contacted every month to receive information about healthy lifestyles. For women in the intervention group, we provided between 2 and 3 l of water per person a day for every 2 weeks. For more detail see Hernandez-Cordero et al. [43]. The original trial was registered at clinicaltrials.gov (NCT01245010).

**Fig. 1:**
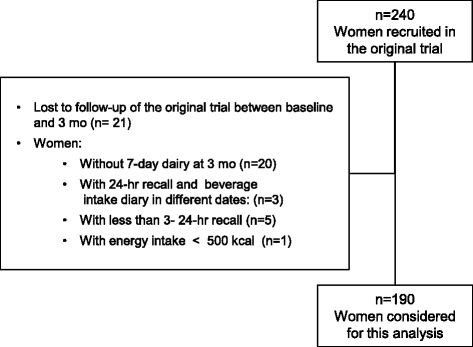
Women included in the validation analysis. Flow diagram of criteria to select women to be included in the current analysis

For the current analysis, we estimated the correlation of reported beverage consumption among three repeated 24-h recalls and one 7-day diary beverage registry, both collected at 3 months of the intervention. Three-month measurements, instead of those at baseline, were used for this analysis given that the 7-day diary was not applied at baseline. We selected those women with data collected with the two instruments in the same week (*n* = 190) (Fig. 1).

The original study was conducted according to the guidelines laid down in the Declaration of Helsinki, and all procedures were approved by the IRB of National Institute of Public Health, Mexico (INSP). Written informed consent was obtained from all subjects.

### Dietary tools and procedures

#### 3-day 24-h recall (24-h recall)

The 24-h recall was administered as an in-person interview consisting in a complete audit of what foods and fluids the participant consumed during the previous 24 h. The interviewer noted the amount consumed, including portion sizes, preparation methods, and recipes for each type of food and fluid reported. Specific extra probes for all fluids (beverages and water) were used in addition to measurement cups to have a better estimation of fluid intake.

The methods to recollect dietary data are described elsewhere [43]. Briefly, three 24-h dietary recalls were administered at 3 months after the original intervention had begun. Highly trained interviewers applied three recalls of three randomly selected nonconsecutive days during one week (two weekdays and one weekend). The first interview took place at the INSP (study headquarters). For the last two, the interviewer visited the participants home to collect the information in the place where food was prepared and obtained the portion sizes as accurately as possible. Each interview lasted approximately 60 min.

#### 7-day diary

This is a self-administrated instrument designed specifically to capture all fluid consumption events. The participants were asked to register all water and beverage consumed during one complete week. The diary included the different meal times (at waking up/before breakfast, during breakfast, during morning/before main meal, during main meal, during afternoon/before dinner, during dinner, and before going to bed), a space to specify the type of beverage, the amount consumed during the day (both in mL and portion sizes), and the brand (in case it was a commercial beverage). To increase the accuracy of the reported portion size, interviewers went over the reported intake with the subjects at every visit using standard measurement cups. In addition, it had a space for interviewers to include the beverage code for the later analysis. The diary was provided to participants in a monthly basis after their visit to the study site. The 7-day diary was given to participants during the same week of the 24-h recall. We provide the actual diary instrument, translated into English, as an additional file to this paper (see Additional file 1).

### Other data collected

Demographic information such age and date of birth, marital status, education level, and wealth possessions and housing conditions was obtained through a questionnaire applied at baseline of the original study.

### Dietary analysis

For local drinks, such as *aguas frescas* (diluted juice with added sugar), *licuados* (smoothies), and *atoles* (hot corn or maize drink), the actual recipe was considered to estimate the energy content of them. The estimation of daily energy intake (kcal/d) from beverages was done using the food composition table compilated by the National Institute of Public Health, USDA, and FNDD Food composition table [44, 45]. The estimation of mililiters per day (mL/d) and kcal/d were average from the three 24-h recall and the 7 day of the registered week for the 7-day diary, for a better approximation to the usual beverage intake.

In addition, we categorized the beverage according to its sugar content and benefits or potential effect on health, based on the Mexican beverage guidance panel [30]. Categories included were:

- *SSB:* Canned or commercial juices; soft drinks; atole (beverage with sugar, without milk); artificial fruit beverages with sugar; sugar-sweetened traditional beverages such as diluted juice with added sugar (lemonade, diluted orange juice), hibiscus water, and rice water (all referred to as aguas frescas); coffee or tea with sugar
- *Fruit juices (100 % juices):* Natural fruit and vegetable juices without added sugar
- *Milk and milk products* without *added sugar:* Milk and drinkable yogurt (plain or light), atole with milk without sugar added, fermented beverage without sugar, coffee or tea with milk or cream substitute without sugar
- *Milk and milk products* with *added sugar:* Sweetened milk, yogurt, or fermented beverages with sugar, atole with milk and sugar, milk with chocolate, milkshake, coffee or tea with milk and sugar added
- *Alcohol beverages:* Beer, alcoholic eggnog, rum, wine, vodka, whisky, with or without other ingredients
- *Unsweetened beverages:* Sugar-free carbonated beverages, atole without sugar nor milk, mineral water, coffee and tea without sugar, beverages with artificial sweetener
- *Plain water*

We present the reported fluid consumption registered with a 24-h recall and diary register in mL and kcal for all and per type of beverages.

### Analysis of socio-demographic characteristics

Age was included in the analysis as a continuous variable, whereas parity, socioeconomic status, education level, profession, and BMI were included as categorical. An indicator of socioeconomic status was developed through principal component analysis, including home appliance ownership (radio, TV set, telephone, refrigerator, stove, etc.), housing characteristics, and number of rooms [46]. Education level was measured as the last year of formal schooling completed and was stratified in to four categories: incomplete middle school and less, complete middle and high school, at least one year of bachelor’s degree, complete bachelor’s degree or more. Even though all women recruited for the original study had a BMI ≥ 25 kg/m^2^, we adjusted the analysis for overweight and obesity, categorizing BMI according to WHO cutoff points (underweight ≤ 18.5, normal 18.5–24.9, overweight 25–29.9 and obese ≥ 30) [47].

Finally, we considered the potential effect of the season when dietary information was collected. We considered two main seasons, spring/summer: April to November; and autumn/winter: December to March.

### Statistical analysis

Potential outliers were explored and reviewed with the actual questionnaires to identify potential data entry errors. Outliers were defined as any intake in grams, mL or kcal more than 4 standard deviations from the mean consumption of the study population. Potential selection bias was explored comparing the women included in this validation study (*n* = 190) and those excluded (*n* = 50).

We verified normality of the data and used the nonparametric statistical analysis when necessary. We estimated the means and standard deviation (or median and 25th and 75th percentile) for total and type of beverage intake (both as energy, kcal/d, and in mL/d) and compared with *t*-test for independent samples or Mann–Whitney rank-sum test. We estimated crude Spearman correlation coefficients to compare the two dietary assessments. We explored whether the allocated intervention group in the original trial affected the comparison of the fluid intake between dietary instruments by comparing the mean kcal/d and mL/d reported by each group, yielding no difference, thus data are presented for the full sample. In addition we estimated the correlation between the beverage 7-day diary and 24-h recall adjusting for potential factors affecting the validation, such as age, BMI, socioeconomic status, education, intervention group, and season when dietary information was collected, using linear regression models. The coefficient of determination of the adjusted model is reported as an estimation of the adjusted correlation. Only those characteristics that were statistically significant or changed 10 % the coefficient were left in the models. Finally, we ranked distributions of intake in mL/d and kcal/d and divide it in tertiles and compare whether individuals were ranked the same with both instruments using Spearman correlation rank. Statistical significance was considered with *p*-values less than 0.05. Data were analyzed using STATA version 10.1 (Stata Corp, TX, US).

## Results

Women included in this validation study (*n* = 190) did not differ in demographic characteristics, socioeconomic status, or weight status (measured as BMI) from women excluded from this analysis (*n* = 50) (data not shown). The subset of women in the validation study were on average 33 y old, 26 % were nulliparous, about 63 % were married or living with someone, and most of them had at least completed high school. The mean BMI was almost 31 kg/m^2^. They were evenly distributed between the two intervention groups (51.6 % in the intervention group and 48.4 in the control group) (Table 1). The total estimated mean energy intake was 1,524 ± 468 kcal/d, of which 16.6 % (253 ± 200 kcal/d) was coming from SSB.

**Table 1:** Selected descriptive characteristics of the sample (*n* = 190)^a^

Age (y)	33.4 ± 6.7
BMI (kg/m^2^)	30.8 ± 3.8
Weight status, *n* (%)^b^	
Normal weight (BMI 18.5–24.9)	12 (6.3)
Overweight (BMI 25.0–29.9)	79 (41.6)
Obese (BMI ≥ 30)	99 (52.1)
Parity	
Nulliparous [*N* (%)]	50 (26.3)
Multiparous [*N* (%)]	140 (73.7)
Marital status [*N* (%)]	
Married/living with someone	119 (62.6)
Not married	71 (37.4)
Education [*N* (%)]	
Incomplete middle school and less	10 (5.3)
Complete high school	83 (43.7)
Technician	31 (16.3)
College or more	66 (34.7)
Occupation [*N* (%)]	
Professional, administrator, or executive	25 (13.2)
Clerical work, administrative support, sales, or technician	76 (40.0)
Crafts, trade, factory work, service, or labor	16 (8.4)
Unemployed, retired, student, or other	73 (38.4)
Dietary intake at 3 months^c^	
Energy intake (kcal/d)	1,524 ± 468
Sugar-sweetened beverage intake (kcal/d)	253 ± 200
Percentage of calories from sugar-sweetened beverages	16.6
Women in the intervention group (%)	98 (51.6)

^a^Mean and standard deviation, or as specified

^b^Based on WHO criteria (47)

^c^From 3-days 24 h-recall

### Estimated median fluid consumption

#### Fluid consumption in mL/d

Compared to 3 days 24-h recall there was no difference on median estimated mL/d registered by the 7-day diary (median: 2,094 mL/d; 25th: 1,700 mL/d; 75th: 2,649 vs. 2,259 mL/d; 25th: 1,710 mL/d; 75th: 2,693 mL/d; *p* = 0.2 for 24-h recall and 7-day diary, respectively) (Table 2). When comparing the fluid intake by type of beverage we found that the reported fluid intake in mL/d was higher (*p* < 0.05) when using 3 days 24-h recall for all type of beverages, except for SSB and water (Table 2), where no differences were seen.

**Table 2:** Fluid reported intake from average of 3-day 24-h recall and 7-day diary registry^a^

Fluid consumption	24-h recall	7-day diary	Absolute difference 24 -h recall vs. 7-day diary (*p*-value)^b^
*Total fluid consumption:*	(*n* = 190)	(*n* = 190)	
kcal/d	266 [152, 375]	311 [187, 430]	−56 (0.072)
mL/d	2094 [1700, 2649]	2259.0 [1710, 2693]	−165 (0.237)
*Type of beverages*			
Sugar sweetened beverages^c^	(*n* = 173)	(*n* = 177)	
kcal/d	126 [76.7, 248.1]	167 [101.8, 268.9]	−41 (0.017)
mL/d	403 [234, 723]	416 [246, 652]	−13 (0.845)
Fruit juices^d^	(*n* = 34)	(*n* = 55)	
kcal/d	63 [39.2, 78.4]	34 [16.8, 48.4]	30 (<0.010)
mL/d	162 [87, 175]	75 [37, 110]	87 (<0.010)
Milk and milk products without added sugar^e^	(*n* = 98)	(*n* = 131)	
kcal/d	60 [39.7, 100.5]	58 [25.5, 106.5]	−2 (0.195)
mL/d	126 [83, 227]	108 [44, 211]	18 (0.025)
Milk and milk products with added sugar^f^	(*n* = 96)	(*n* = 113)	
kcal/d	92 [59.9, 152.5]	73 [33.5, 129.5]	19 (0.011)
mL/d	150 [87, 264]	87 [48, 177]	63 (<0.010)
Alcohol beverages^g^	(*n* = 30)	(*n* = 49)	
kcal/d	95 [45.6, 159.4]	45 [27.5, 110.2]	50 (<0.010)
mL/d	133 [106, 300]	90 46, 157]	43 (<0.010)
Unsweetened beverages^h^	(*n* = 74)	(*n* = 85)	
kcal/d	3 [1.6, 13.5]	2 [0.8, 9.1]	1 (0.082)
mL/d	190 [86, 370]	143 [71, 339]	47 (0.023)
Water	(*n* = 187)	(*n* = 190)	
mL/d	1340 [883, 1917]	1554 [821, 1986]	−214 (0.261)

^a^Median and 25th–75th percentile, or as specified

^b^Mann–Whitney rank-sum test

^c^Sugar-sweetened beverages: canned or commercial juices, soft drinks, *atole* (beverage with sugar, without milk), artificial fruit beverages with sugar, sugar-sweetened traditional beverages, such as lemonade, hibiscus water, and rice water [*aguas frescas*], coffee or tea with sugar

^d^Fruit juices: natural fruit and vegetables juices without sugar added

^e^Milk and milk products without added sugar: milk and drinkable yogurt (plain or light), atole with milk, without sugar added, fermented beverage without sugar, coffee or tea with milk or cream substitute

^f^Milk and milk products with added sugar: sweetened milk, yogurt or fermented beverages, atole with milk and sugar, milk with chocolate, milkshake, coffee or tea with milk and sugar added

^g^Alcohol beverages: beer, alcoholic eggnog, rum, wine, vodka, whisky, with or without other ingredients

^h^Unsweetened beverages: sugar-free carbonated beverages, atole without sugar or milk, mineral water, coffee and tea without sugar, beverages with noncaloric sweeteners

#### Fluid consumption in kcal/d

Median estimated energy from total beverage consumption was not statistically significant different between the two dietary assessment tools (24-h recall median: 266 kcal/d; 25th: 152 kcal/d, 75th: 376 kcal/d; 7-day diary median: 311 kcal/d, 25th: 187 kcal/d, 75th: 430 kcal/d; *p* = 0.07). By type of beverages, the reported consumption was higher when using the 24-h recall for all type of beverages (*p* < 0.05), except for SSB and beverages without added sugars (milk and milk products and unsweetened beverages). For the last, the difference was not statistically different, and for SSB the reported energy intake was actually lower for 24-h recall than for the 7-day diary (Table 2).

### Correlation analysis

The crude correlation among the two dietary instruments was highly significant for total fluid consumption in mL/d (*r* = 0.73; *p* < 0.001). By type of fluid, the range of the Spearman correlation coefficient was *r* = 0.4 (*p* < 0.001) for fluid included in the milk and milk products without added sugar to *r* = 0.8 for water (*p* < 0.001). SSB and alcohol correlation were the second and third, respectively, higher correlation coefficients. The correlation between the two instruments was not statistically significant for 100 % fruit juices (Table 3). For fluid intake measured as energy (kcal/d), the correlation coefficient was similar to that for mL (*r* = 0.74; *p* < 0.001). All the correlation coefficients between the two dietary methods were statistically significant, except for reported fruit juices (Table 3). The highest correlation coefficient was for alcoholic beverages (*r* = 0.78, *p* < 0.001), followed by SSB (*r* = 0.73, *p* < 0.001).

**Table 3:** Correlation of beverage reported intake from average of 3-day 24-h recall and 7-day diary registry

	CC^a^
*Total fluid consumption:*	(*n* = 190)
kcal/d	0.743***
mL/d	0.737***
*Type of beverages*	
Sugar-sweetened beverages	(*n* = 171)
kcal/d	0.726***
mL/d	0.786***
Fruit juices	(*n* = 26)
kcal/d	0.292
mL/d	0.328
Milk and milk products without added sugar	(*n* = 91)
kcal/d	0.483***
mL/d	0.400***
Milk and milk products with added sugar	(*n* = 83)
kcal/d	0.437***
mL/d	0.484***
Alcohol beverages	(*n* = 24)
kcal/d	0.776***
mL/d	0.757***
Noncaloric beverages	(*n* = 66)
kcal/d	0.568***
mL/d	0.601***
Water	(*n* = 187)
mL/d	0.850***

^a^Spearman correlation

***p <0.001

From the cross tabulated tertiles of the 24-h recall and 7-day diary we found a high rank correlation (*r* = 0.65, *p* < 0.001 for both mL/d and kcal/d) for total fluid intake (Additional file 2). For fluid consumption in mL the categories of water (*r* = 0.78, *p* < 0.001), SSB (*r* = 0.69, *p* < 0.001), alcohol beverages (*r* = 0.62, *p* = 0.001), and noncaloric beverages (*r* = 0.55, *p* < 0.001) had a highly significant rank correlation, indicating an agreement between ranking. The lowest rank correlation (*r* < 0.5) was seen for milk and milk products with added sugars, and milk and milk products without added sugars (Additional file 3).

For fluid intake reported as energy (kcal/d) by type of beverage, as for the beverage consumption expressed in mL/d, the highest rank correlation coefficients were for alcoholic beverages (*r* = 0.69, *p* < 0.001) and SSBs (*r* = 0.61, *p* < 0.001) ((Additional file 3). It is important to notice that the rank correlation of fruit juices was no significant neither in mL/d or kcal/d.

The adjusted correlation, measured through linear regression, between 24-h recall and 7-day diary, adjusting for age, BMI, education, socioeconomic status, and intervention group remained highly significant for total beverage consumption in mL/d (*R*^2^ = 0.65, *p* < 0.001), water (*R*^2^ = 0.72, *p* < 0.001), and SSB (*R*^2^ = 0.65, *p* < 0.001). All other type of beverages had a statistically significant *R*^2^ values (*p* < 0.05), but were lower than 0.5 (Table 4), except for juices, which was not statistically significant. For fluid intake expressed in kcal/d, the correlation remained highly significant for alcoholic beverages (*R*^2^ = 0.62, *p* ≤ 0.001) and SSBs (*R*^2^ = 0.50, *p* ≤ 0.001).

**Table 4:** Adjusted correlation of beverage intake from average of 3-day 24-h recall and 7-day diary registry^a^

	*R* ^2^	Root SME	*p* (model)
*Total beverage consumption:* (*n* = 190)			
kcal/d	0.522	581.9	<0.001
mL/d	0.650	424.6	<0.001
*Type of beverages*			
Sugar-sweetened beverages (*n* = 171)			
kcal/d	0.503	425.3	<0.001
mL/d	0.655	239.5	<0.001
Fruit juices (*n* = 26)			
kcal/d	0.164	147.1	0.745
mL/d	0.213	78.7	0.531
Milk and milk products without added sugar (*n* = 91)			
kcal/d	0.153	201.7	0.005
mL/d	0.156	102.9	0.004
Milk and milk products with added sugar (*n* = 83)			
kcal/d	0.318	329.1	<0.001
mL/d	0.228	115.2	<0.001
Alcohol beverages (*n* = 24)			
kcal/d	0.620	437.4	0.002
mL/d	0.375	150.0	0.044
Noncaloric beverages (*n* = 66)			
kcal/d	0.103	119.6	0.472
mL/d	0.412	156.3	<0.001
Water (*n* = 187)			
mL/d	0.723	354.3	<0.001

^a^Linear regression models. Coefficient of determination (*R* ^2^) of each model is used as an estimator of the adjusted correlation. Adjusted by: age, BMI, education, socioeconomic status (tertiles), and intervention group

## Discussion

This study demonstrated no significant difference in the estimated total median volume of fluid intake, expressed as mL/d between a fluid 7-day diary and the 3-day 24-h recall as the reference dietary method; however, there were significant differences in mL/d and kcal/d when analyzed by beverage categories. There were highly significant crude correlation coefficients for total fluid intake, showing a stronger correlation when reporting water, alcohol, and SSB intake. The more meaningful rank correlations were for fluid intake in mL/d: water, alcohol beverages, and SSB and for fluid intake in kcal/d: alcohol beverages and SSB (rank correlation ≥ 0.6). When adjusted for age, BMI, education, socioeconomic status, and intervention group, the correlation was still high and significant for total fluid consumption in mL/d, water, and SSB (*R*^2^ ≥ 0.6, *p* < 0.001). Hedrick and colleagues report similar results in a cross-sectional study in healthy U.S. adults aged >21 years, determining the validity and reliability of a self-administered beverage intake questionnaire, which estimates mean daily intake of beverage consumption (g, kcal) across several beverages categories [37]. The beverage intake questionnaire showed a high correlation vs. a 4-day food intake record in daily consumption (g) of water, total beverages, and SSB. To our knowledge, there is no specific validated dietary instrument to collect fluid intake in Mexican populations.

One potential explanation of the energy intake differences in our study is the methodology of each dietary tool in the registration of detailed information about added sugar. For the 3-day 24-h recall, well-trained interviewers asked the amount of sugar added to the beverages. On the other hand the 7-day diary was a self-administered questionnaire, and in spite of the meticulous review of the questionnaires once women handed them back, when it was not specified whether beverage had added sugar or there was not an exact amount of it, it was assumed that they were SSB and a standard sugar portion was assumed for energy estimations. This hypothesis is supported by the results of beverages without added sugars. When comparing the reported energy between the two instruments, these two categories were the only ones with no statistically significant differences. The correct measurement of added sugar in beverages is particularly important in Mexico where 100 % fruit juices with water and added sugars, often called aguas frescas, represent a major component of caloric beverage intake for all age groups and added sugar is important in coffee and 100 % juices also [12–14].

The difference on beverage intake in mL/d by type of beverage might be explained by the reported portion size. As already stated, the main difference between instruments, besides the temporality, was that the dietary information with 3-day 24-h recall was collected by well-trained interviewers with special care on portion sizes, versus the 7-day diary self-administered questionnaire.

The notable benefit of the 7-day diary was in the one kind of beverage that has received great attention in Mexico, SSB. The schools ban or limit them since 2010 [48], and recently the government approved a 10 % tax on SSBs, with the purpose of discouraging and decrease SSB consumption among the population [31]. As a less healthful beverage, research from other countries might suggest recall of SSB and other food with high energy, sugar, and fat content would be underestimated systematically [49–52]. The highly significant crude and adjusted correlation coefficients of the 7-day diary to the reference method on this study (24-h recall) is indicative of one potential strength of the 7-day diary to measure SSB intake.

The major weakness of this study is the lack of an ideal gold standard method to record fluid intake of free-living individuals. So far, 24-h urine is one of the best markers of fluid intake in subjects with normal renal function [42]; however, it has some limitations. For individuals who perspire in abundance (i.e., active persons and athletes), 24-h urine is not an accurate marker because it does not take into account water losses via perspiration and feces [53], and 24-h urine sample collection represents a burden for participants. In this study we used as a reference measure repeated 24-h recalls. Even though this is considered an adequate reference [54], it is important to mention that it also has some limitations. The 24-h recall has inherent errors from memory, portion size estimations, and distortions of reported dietary recall, resulting in potential loss of validity as a reference method.

The fact that we used the reported and registered beverage intake from the 24-h recall and 7-day diary, respectively, after 3 months of the beginning of the intervention of the original trial might affect both internal and external validity of the study. However, there were no differences between the intervention and control groups for either total beverage intake or any beverage category (data not shown). We adjusted the regression analysis for the intervention group. For external validity, women in the original trial, even those in the control group, were receiving general information of healthy life styles and were contacted every month. The fact that women received this information and the continuous contact with the personnel from the clinical trial may have resulted in a group of women more conscious about their eating and drinking habits than the general population, thus having a better report and register of beverages and diet in general. In addition, for external validity, the fact that our study included only overweight and obese women with a high intake of SSB (>250 kcal/day) limits the generalization of the results to women with same characteristics. Furthermore, it is well recognized that the prevalence of underreporting of energy consumption and certain foods and beverages considered as “unhealthy” is higher in women and even more among overweight and obese women compared to men and normal weight women, respectively [55, 56]. One other limitation to this study was the fact that the 7-day diary was provided to women during the same week as the 24-h recall. The sequence of measurement in this study might have resulted in an increased correlation between both instruments [57]. To confirm our results, a study evaluating the methodologies in a design where subjects will alternatively filled a 3-day 24-h recall or a 7-day diary, in a randomized manner, with a “wash-out” period between the two fillings would be more appropriate.

The study has important strengths. Our sample size (*n* = 190 women) and number of repeated measures were appropriate, considering that a sample size of 110 subjects with at least two replicates of 24-h recall and 2 to 4 days of diet recording will provide adequate statistical power and remove the within-person variation. Overall, studies using 3 days of repeated measured per subject, 200 subjects is adequate for validation studies [54]. We evaluated potential selection bias by comparing women included in this analysis with those recruited for the original trial. There were no differences in age, marital status, or other demographic characteristics (data not shown).

## Conclusions

Overall, the 7-day diary showed high and strong rank correlations with that reported in the 24-h recall, suggesting that the diary method is a valid tool to evaluate total fluid, water, and SSB intake in this population, but not all individual beverage categories. The gaps between mL/d and kcal/d for the key sources of caloric beverages suggest that further refinements may be useful. This could include improved participant training and more conscientious review by the field workers. Diet recording methods are appropriate instruments that do not depend on memory, so the measurement error, when used in literate and cooperative populations, might be reduced. However, there is always the problem that this instrument may influence the actual fluid intake [55]. As caloric beverages intake increase across the globe, as well as the strategies to discourage the intake of these beverages along with the water intake promotion, accurate methods to assess beverage intake are needed. Dietary tools such as the 7-day diary might be one approach for data collection for large-scale samples with similar characteristics to our study population (overweight and obese women with high intake of sugar-sweetened beverages).

## Additional files

Additional file 1:
**Beverage Diary Register.** The 7-day diary instrument used in the study, translated into English (PDF 102 kb) Additional file 2:
**Cross classification and rank correlation by tertiles.** Total fluid intake (mL/d and kcal/d) cross-classification and rank correlation by tertiles (*n* = 190). Cross classification and rank correlation of the 24 h-h recall and 7-day diary. (PDF 146 kb) Additional file 3:
**Spearman rank correlation.** Spearman rank correlation (RC) by type of beverage (mL/d and kcal/d). (PDF 152 kb)

## Abbreviations

BMI: Body mass index
FFQ: Food frequency questionnaire
kcal/d: Kilocalories per day
mL/d: milliliters per day
SSB: Sugar-sweetened beverage
WHO: Word Health Organization
