# Supplementary material for: Comparing a 7-day diary vs. 24 h-recall for estimating fluid consumption in overweight and obese Mexican women
Source: BMC Public Health. 2015 Oct 7;15:1031. doi: 10.1186/s12889-015-2367-0 (PMC4597614; doi:10.1186/s12889-015-2367-0)
Supplement: Additional file 1: — Beverage Diary Register. The 7-day diary instrument used in the study, translated into English (PDF 102 kb) [file 12889_2015_2367_MOESM1_ESM.pdf]

Additional file 1.Comparing a 7-days diary vs. 24 hr-recall for estimating fluid consumption in overweight and obese Mexican women.

Beverage Diary Register

Beverage Diary Register STAGE______________

ID |__|__|__|__|__|__|__| NAME ____

M T W T F S S DATE _____/____/_____

	BEVERAGE NAME	CODE (INTERVIEWER ONLY)	PORTION SIZE	TOTAL AMOUNT	COMERCIAL BRAND
AT WAKING UP / BEFORE BREAKFAST					
DURING BREAKFAST					
BEFORE LUNCH					
DURING LUNCH					
DURING THE EVENING / BEFORE DINNER					
DURING DINNER					
BEFORE GOING TO BED					
