# Supplementary material for: Comparing a 7-day diary vs. 24 h-recall for estimating fluid consumption in overweight and obese Mexican women
Source: BMC Public Health. 2015 Oct 7;15:1031. doi: 10.1186/s12889-015-2367-0 (PMC4597614; doi:10.1186/s12889-015-2367-0)
Supplement: Additional file 2: — Cross classification and rank correlation by tertiles. Total fluid intake (mL/d and kcal/d) cross-classification and rank correlation by tertiles (n = 190). Cross classification and rank correlation of the 24 h-h recall and 7-day diary. (PDF 146 kb) [file 12889_2015_2367_MOESM2_ESM.pdf]

Additional file 2.Comparing a 7-days diary vs. 24 hr-recall for estimating fluid consumption in overweight and obese Mexican women.

Total fluid intake (mL/d and kcal/d) cross-classification and rank correlation by tertiles (*n* = 190)

	**24-hr recall tertile (mL/d)**			
**7-day diary tertile (mL/d)**	**1 (477–1917)**	**2 (1921–2502)**	**3 (2503–3965)**	**Total**
**1 (643–1910)**	47	16	1	64
**2 (1938–2580)**	10	35	18	63
**3 (2586–5078)**	7	12	44	63
**Total**	64	63	63	190
Spearman rank correlation, $r = 0.654$, $p < 0.001$				

	**24-hr recall tertile (kcal/d)**			
**7-day diary tertile (kcal/d)**	**1 (0–840)**	**2 (846–1449)**	**3 (1455–5300)**	**Total**
**1 (0–900)**	47	14	3	64
**2 (895–1624)**	13	32	18	63
**3 (1633–4346)**	4	17	42	63
**Total**	64	63	63	190
Spearman rank correlation, $r = 0.646$, $p < 0.001$				
