# Supplementary material for: Comparing a 7-day diary vs. 24 h-recall for estimating fluid consumption in overweight and obese Mexican women
Source: BMC Public Health. 2015 Oct 7;15:1031. doi: 10.1186/s12889-015-2367-0 (PMC4597614; doi:10.1186/s12889-015-2367-0)
Supplement: Additional file 3: — Spearman rank correlation. Spearman rank correlation (RC) by type of beverage (mL/d and kcal/d). (PDF 152 kb) [file 12889_2015_2367_MOESM3_ESM.pdf]

Additional file 3.Comparing a 7-days diary vs. 24 hr-recall for estimating fluid consumption in overweight and obese Mexican women.

Spearman rank correlation (RC) by type of beverage (mL/d and kcal/d)

**Type of beverage**	**mL/d**		**Kcal/d**	
	**RC**	***p*-value**	**RC**	***p*-value**
Sugar-sweetened beverages (SSB)[1] (*n* = 171)	0.693	<0.001	0.614	<0.001
Fruit juices[2] (*n* = 26)	0.297	0.140	0.297	0.140
Milk and milk products without added sugars[3] (*n* = 91)	0.295	0.004	0.377	<0.001
Milk and milk products with added sugars[4] (n = 83)	0.344	0.001	0.345	0.001
Alcohol beverages[5] (*n* = 24)	0.625	0.001	0.687	<0.001
Noncaloric beverages[6] (*n* = 66)	0.545	<0.001	0.539	<0.001
Water (*n* = 187)	0.776	<0.001	—	—

[1] Sugar sweetened beverages: canned or commercial juices, soft drinks, *atole* (beverage with sugar, without milk), artificial fruit beverages with sugar, sugar-sweetened traditional beverages, such as lemonade, hibiscus water, and rice water [*aguas frescas*], coffee or tea with sugar.

[2] Fruit juices: natural fruit and vegetables juices without sugar added.

[3] Milk and milk products without added sugar: milk and drinkable yogurt (plain or light), atole with milk, without sugar added, fermented beverage without sugar, coffee or tea with milk or cream substitute.

[4] Milk and milk products with added sugar: sweetened milk, yogurt or fermented beverages, atole with milk and sugar, milk with chocolate, milkshake, coffee or tea with milk and sugar added.

[5] Alcohol beverages: Beer, alcoholic eggnog, rum, wine, vodka, whisky, with or without other ingredients.

[6] Unsweetened beverages: sugar-free carbonated beverages, atole without sugar nor milk, mineral water, coffee and tea without sugar, beverages with noncaloric sweetener.
